# Mucin Biology as a Local Diagnostic and Promising Therapeutic Target in Endometriosis: Expression and Glycosylation Profiling

**DOI:** 10.3390/ijms27021010

**Published:** 2026-01-20

**Authors:** Renata V. Velho, Christoph Schüßler, Lisa Strey, Stefanie Weigel, Susanne Thomsen, Franziska Ebert, Jonathan Pohl, Sylvia Mechsner, Maria Maares

**Affiliations:** 1 Department of Gynecology Charité with Centre of Oncological Surgery, Endometriosis Research Centre Charité, Campus Virchow-Klinikum, Augustenburger Platz 1, 13353 Berlin, Germany; renata.voltolini-velho@charite.de (R.V.V.); lisa.strey@charite.de (L.S.); 2 Department of Food Chemistry, Institute of Nutritional Science, University of Potsdam, Karl-Liebknecht-Str. 24-25, 14476 Potsdam, Germany; christoph.schuessler@uni-potsdam.de (C.S.); stefanie.weigel@uni-potsdam.de (S.W.); fraebert@uni-potsdam.de (F.E.); 3 Department of Food Chemistry and Toxicology, Technische Universität Berlin, Strasse des 17. Juni 135, 10623 Berlin, Germany; susanne.thomsen@tu-berlin.de; 4 Institute of Pathology, Charité-Universitätsmedizin Berlin, Charitéplatz 1, 10117 Berlin, Germany; jonathan.pohl@charite.de

**Keywords:** endometriosis, mucins, glycosylation, lesion pathophysiology

## Abstract

Endometriosis (EM) is a chronic inflammatory disease characterized by the growth of endometrial-like tissue outside the uterus, yet its molecular mechanisms remain poorly understood. This study investigated the expression of mucins (MUC1, MUC2, MUC5AC, MUC6, MUC16) and their *O*-glycans in endometriotic lesions, given their roles in epithelial protection, adhesion, and immune modulation. Using immunohistochemistry, Western blotting, lectin profiling, histochemical staining, and transcriptomic analysis, we compared mucin levels and glycosylation patterns in eutopic and ectopic tissues from women with and without endometriosis and measured mucin-derived tumor markers in serum (CA 125/MUC16 and CA 15-3/MUC1) and peritoneal fluid (CA 125/MUC16). The results showed significant upregulation of all mucins in EM biopsies, with increased MUC1 transcript levels, while MUC6 and MUC16 protein levels did not always align with transcripts. Yet, tumor markers CA 125 and CA 15-3 showed no significant differences between groups. Looking at mucin distribution in biopsies of peritoneal (pEM), deep infiltrating and ovarian EM, MUC1 was significantly overexpressed in lesions of all EM forms, while MUC5AC was significantly elevated in pEM. Lectin analysis revealed specific glycan changes, including elevated core-1 *O*-glycans and α(1-2)-linked fucosylation, while sialylation remained unchanged. These findings demonstrate consistent mucin dysregulation and glycan alterations, implicating their roles in epithelial adhesion, immune evasion, and lesion persistence. Mucin biology thus emerges as a promising target for diagnostic and therapeutic strategies in endometriosis.

## 1. Introduction

Endometriosis is an estrogen-dependent inflammatory disorder, a chronic and debilitating condition affecting about 10% of women of reproductive age, with profound consequences for reproductive health and quality of life [1,2,3]. It is characterized by the ectopic implantation and growth of endometrial-like epithelial and stromal cells outside the uterine cavity, predominantly on pelvic organs such as the ovaries, fallopian tubes, and peritoneum [3,4,5]. The disease manifests with a spectrum of clinical symptoms, including chronic pelvic pain, dysmenorrhea, and infertility, yet its diagnosis is frequently delayed by several years due to the heterogeneity of symptoms and the lack of non-invasive, reliable diagnostic tools [2,6]. Early and accurate diagnosis is crucial, not only to improve patient care but also to limit disease progression and its long-term consequences [2,3]. Hence, there is an urgent need to identify molecular markers that can enable early diagnosis and provide insights into the mechanisms underlying disease pathogenesis [7].

One promising avenue of research centers on alterations in glycosylation, a post-translational modification critical for protein function, stability, and cell–cell interactions [8,9]. Among glycoproteins, mucins represent a key family implicated in various pathological processes. Mucins are high molecular weight, heavily *O*-glycosylated glycoproteins that form protective mucous barriers on epithelial surfaces and play vital roles in cell signaling, adhesion, and immune modulation [10,11]. The extensive glycan chains on mucins constitute up to 80% of their mass and mediate interactions with the extracellular environment, including the extracellular matrix and immune cells [10]. Aberrant mucin expression and altered glycosylation patterns have been documented in diverse disease states, including chronic inflammation and malignancies, where they contribute to altered cell adhesion, immune evasion, and enhanced invasiveness [12,13,14,15].

In the context of endometriosis, emerging evidence suggests that mucins, in particular MUC1, MUC2, MUC5AC, MUC6, and MUC16 [8,16,17], and their glycosylation patterns undergo significant changes that may directly influence disease progression. In normal endometrium, mucins maintain tissue integrity and contribute to immune homeostasis. However, in ectopic endometrial lesions, altered mucin expression and aberrant glycosylation may disrupt these functions, facilitating the invasive behavior of endometrial cells and enabling them to escape immune detection [16]. Specifically, changes in glycan structures on mucins may mask antigenic epitopes or modulate the interaction between endometrial cells and immune effector cells, thereby promoting lesion persistence and chronic inflammation. These molecular alterations may also impact cell adhesion dynamics, contributing to the attachment and survival of endometrial tissue at ectopic sites [16,18].

Given the potential of mucins and their *O*-glycosylation patterns as both biomarkers and therapeutic targets, this study aims to elucidate their roles in endometriosis pathogenesis. By characterizing the specific changes in mucin expression and *O*-glycosylation in ectopic lesions compared with eutopic tissue, we aim to advance understanding of the molecular mechanisms underlying endometriosis. Ultimately, this knowledge could pave the way for the development of novel diagnostic tools and targeted therapies aimed at improving patient outcomes in this debilitating disease.

## 2. Results

### 2.1. Dysregulated Mucin Expression in Endometriosis Lesions

**Figure 1:**
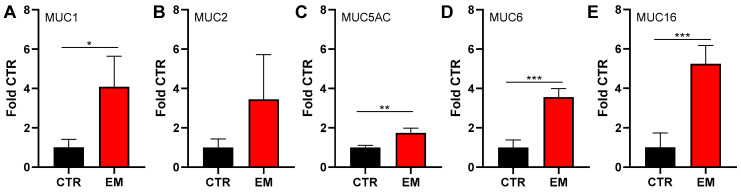
Mucin expression in endometriosis biopsies. Protein expression of MUC1 (**A**), MUC2 (**B**), MUC5AC (**C**), MUC6 (**D**), and MUC16 (**E**) was analyzed by agarose gel Western blotting in cryo-conserved biopsies obtained from endometriotic lesions located in the fossa ovarica, Douglas pouch, and pelvic wall. Peritoneal tissue from donors without endometriosis served as control (CTR). Data are presented as mean values + SD from N = 5 endometriosis patients and N = 3 CTR donors. Statistical differences were assessed using unpaired *t*-tests (* *p* < 0.05; ** *p* < 0.01; *** *p* < 0.001). Representative blots are presented in Supplementary Figure S1.

Using cryo-conserved biopsies of endometrial lesions obtained during laparoscopy, mucin protein expression was assessed by agarose gel Western blotting. We included transmembrane mucins MUC1 and MUC16, as well as secreted and gel-forming mucins MUC2, MUC5AC, and MUC6. Due to the limited amount of sample material and the availability of commercially available and reliable mucin antibodies, not all known 20 human mucins were examined [10]. Instead, the focus was placed on those that had previously been detected in healthy endometrium, in endometrial transformations, or in malignant tumors of the endometrium [8,16,17]. All tested mucins were upregulated in endometriosis tissue biopsies compared with CTRs (Figure 1, Supplementary Figure S1). These findings indicate that mucin production is broadly increased in endometriotic lesions, independent of anatomical localization.

**Figure 2:**
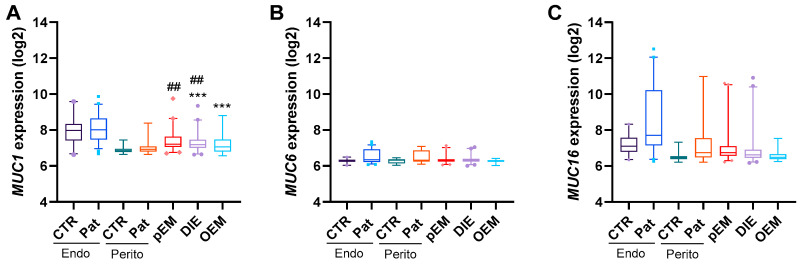
Mucin gene expression in endometriosis (EM) lesions. Gene expression of *MUC1* (**A**), *MUC6* (**B**), and *MUC16* (**C**) in tissues from endometriosis (EM) patients and control (CTR) groups was analyzed using the EndometDB Turku Endometriosis Database (https://endometdb.utu.fi/ (accessed on 3 July 2024). For this, data from patients with peritoneal EM (pEM, N = 76), deep infiltrating EM (DIE, N = 86), and ovarian EM (OEM, N = 28) as well as mucin expression in endometrium (Endo) and peritoneum (Perito) of CTR and patients (Pat.) were included. Results are shown as boxplots, and significant differences between EM and CTRs were tested with Kruskal–Wallis followed by Dunn’s multiple comparison test (vs. endometrium (Endo) CTR *** *p* < 0.001; vs. peritoneum (Perito) CTR ## *p* < 0.01). Data are shown as whiskers plot with interquartile range.

Gene expression data obtained from the EndometDB Turku Endometriosis Database provided additional insight into the transcriptional regulation of these glycoproteins. Among the genes analyzed, *MUC1* was significantly increased in endometriotic lesions compared with control peritoneum, although expression remained lower than in healthy endometrium (Figure 2A). In contrast, the transcript levels of *MUC6* and *MUC16* did not differ significantly between patients and controls (Figure 2B,C). No gene expression data were available for MUC2 or MUC5AC in this dataset, leaving their transcriptional contribution unresolved.

**Figure 3:**
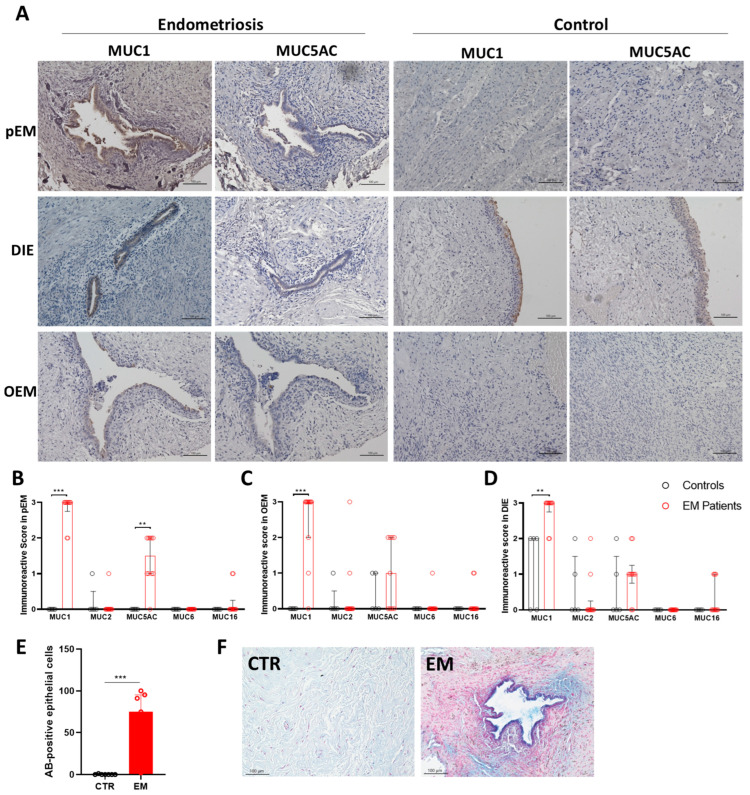
Mucin expression and distribution in endometrial lesions. Shown are representative images of immunohistochemical staining of peritoneal endometriosis (pEM) in the fossa ovarica, deep infiltrating endometriosis (DIE) in the bladder, ovarian endometriosis (OEM) and control tissue with antibodies against human MUC1 or human MUC5AC (brown color), nuclei were stained with hematoxylin (blue color). As controls, tissue from peritoneum, ovaries and bladder were stained. (**A**). Expression of MUC1, MUC2, MUC5AC, MUC6, and MUC16 in endometrial lesions of peritoneal endometriosis (pEM, (**B**)), deep infiltrating endometriosis (DIE, (**C**)) and ovarian endometriosis (OEM, (**D**)) was analyzed with immunohistochemical staining. Results are presented as immunoreactive score (IRS) of endothelial cells from endometriosis lesions. Alcian blue (AB)-positive epithelial cells in CTR and EM tissue were detected with AB-staining (**E**,**F**). Scale Bar = 100 µm. Results are shown as median and interquartile range. Statistical differences were tested with Mann–Whitney-Test (** *p* < 0.01; *** *p* < 0.001).

Immunohistochemical staining of endometriotic lesions provided spatial resolution of mucin distribution within the tissue. Representative immunostaining of deep infiltrating endometriosis (DIE) in the bladder, peritoneal endometriosis (pEM) in the fossa ovarica, and ovarian endometriosis (OEM) revealed intense staining for MUC1 and MUC5AC in epithelial cells of endometrial glands. In contrast, stromal compartments remained largely negative (Figure 3A). As control tissues, the unaffected ovary, peritoneum, bladder, and rectum were stained with the respective mucin antibodies (Figure 3A). Semi-quantitative evaluation using the immunoreactive score (IRS) highlighted significant differences between mucin subtypes and lesion types (Figure 3B–D). MUC1 was consistently upregulated across all lesion subtypes. MUC2 showed only a modest increase, confined mainly to ovarian lesions (Figure 3D), and was characterized by high inter-individual variability. MUC5AC expression was significantly elevated in pEM, whereas MUC6 and MUC16 did not show localized increases in lesion epithelium, despite their overall elevation in tissue lysates. Alcian blue staining further confirmed a significant increase in acidic glycoproteins in epithelial cells from EM biopsies (Figure 3E,F).

### 2.2. Unaffected Serum and Peritoneal Fluid Tumor Markers in Endometriosis

**Figure 4:**
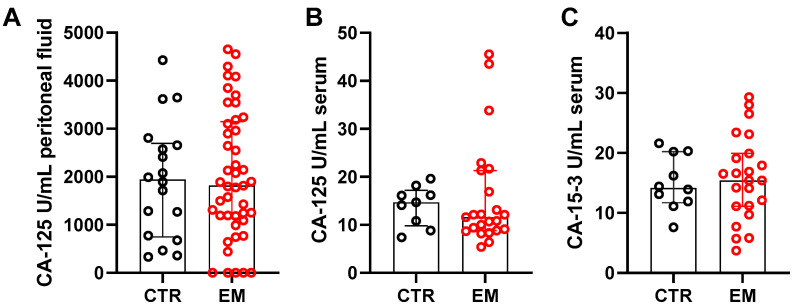
Tumor markers in serum and peritoneal fluid of endometriosis patients. Concentration of CA 125 (MUC16) (**A**,**B**) and CA 15-3 (MUC1) (**C**) in peritoneal fluid (**A**) and in serum (**B**,**C**) of patients without (control, CTR) and with endometriosis (EM), measured with an electrochemiluminescence immunoassay (ECLIA). Statistical significance between EM and CTR was tested with the Mann–Whitney U test. Data are presented as median and interquartile range (peritoneal fluid EM: N = 44; CTR: N = 18; serum EM: N = 22; CTR: N = 10).

The cleaved extracellular domain of MUC16 is commonly measured in the clinic under the designation CA 125, and the soluble cleaved fragment from the transmembrane mucin MUC1 is measured as CA 15-3, another tumor-associated soluble mucin marker. Thus, we assessed their levels in both the peritoneal fluid and serum of endometriosis patients (all endometriosis types). Surprisingly, none of the markers showed significant differences between patients and controls (Figure 4). We also aimed to determine whether CA 125 levels were specifically elevated in patients with ovarian endometriosis. However, only six patients had ovarian endometriosis (which included cases isolated or in combination with peritoneal and/or deep infiltrating endometriosis), and there was no statistical significance found for the peritoneal fluid and serum (*p* = 0.172; *p* = 0.942, respectively). Median serum CA 125 levels were slightly lower in endometriosis patients, whereas CA 15-3 showed greater variability within the EM group. It is important to note that, in the control group, of the 18 controls analyzed for peritoneal fluid, only 4 had non-endometriosis cysts, and among the 10 controls for serum, none had cysts. CA 125 and CA 15-3 values did not differ between controls with or without cysts. Of note, CA 125 concentration in serum and peritoneal fluid did not correlate (r = 0.183; *p* = 0.393).

### 2.3. Altered Glycosylation Pattern in Endometriosis Biopsies

**Figure 5:**
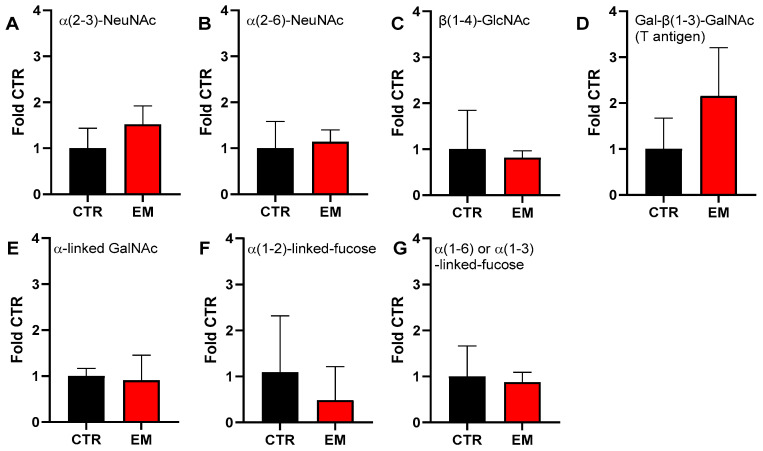
Affected glycosylation pattern in endometriosis biopsies. Glycans were analyzed by agarose gel Western blotting and glycan-specific lectins in cryo-conserved biopsies obtained from endometriotic lesions located in the fossa ovarica, Douglas pouch, and pelvic wall. For this, α(2-3)-N-acetylneuraminic acid (NeuNAc) was detected with Maackia Amurensis lectin (MAL) II (**A**), NeuNAc in α(2-6)-linkage to terminal galactose with Sambucus Nigra lectin (SNA) (**B**), β(1-4)-linked N-Acetal-D-glucosamin (GlcNAc) with wheat germ agglutinin (WGA) (**C**), galactose in β(1-3)-linkage to N-Acetly-D-galactosamin (GalNAc) with peanut agglutinin (PNA) (**D**), α-linked GalNac with Dolichos Biflorus agglutinin (DBA) (**E**), fucose in α(1-2) linkage with Ulex Europaeus agglutinin (UEA) I (**F**), and fucose either α(1-6) linked to N-Acetyl-D-glucosamine or α(1-3) linked to N-Acetyllactosamine with Aleuria Aurantia lectin (AAL) (**G**). Peritoneal tissue from donors without endometriosis served as control (CTR). Shown are MW + SD from N = 5 endometriosis patients and N = 3 CTR donors. Representative blots are shown in Supplementary Figure S2.

Given the importance of glycosylation for the biological function of mucins, we next investigated glycan profiles of EM tissue biopsies using lectin-based agarose gel Western blotting, which enabled the separation and analysis of high-molecular-weight proteins (Figure 5A–G, Supplementary Figure S2).

While overall sialylation, assessed with MAL II and SNA (Figure 5A,B), did not differ between patients and controls, a trend for increased glycan substitution in EM tissue was only observed for galactose in β(1-3)-linkage to GalNAc (T-antigen, core-1 *O*-glycan) (Figure 5D). In contrast, α-linked GalNAc detected by DBA did not differ between groups and showed wide inter-individual variation (Figure 5D). Fucosylation detected by AAL, staining fucose either α(1-6) linked to N-Acetyl-D-glucosamine or α(1-3) linked to N-Acetyllactosamine, was unchanged, whereas UEA I reactivity indicated a slight decrease in α(1-2)-fucosylation in EM tissues and higher variation in CTR samples.

### 2.4. Correlation with Clinical Data

Correlation analyses of the immunoreactive scores of MUC1, MUC2, MUC5AC, MUC6, or MUC16 with clinical data (age, BMI, EM type, revised American Society for Reproductive Medicine (rASRM) stage, menstrual cycle phase, gravidity, parity, infertility) revealed no significant associations (Supplementary Table S1). Similarly, tumor marker concentrations in serum and peritoneal fluid did not correlate with pain symptoms or disease classification (Supplementary Table S2).

## 3. Discussion

In this study, we demonstrate that mucin expression and glycosylation are profoundly altered in endometriotic lesions, underscoring their potential role in disease pathophysiology. By combining immunochemical, histochemical, and lectin-based analyses with transcriptomic data and tumor marker quantification, we provide a comprehensive overview of mucin regulation in endometriosis. Our results revealed a broad upregulation of mucins in endometriosis lesions compared with control peritoneal tissue. This included both transmembrane mucins, such as MUC1 and MUC16, and secreted gel-forming mucins, such as MUC2, MUC5AC, and MUC6. Previous work suggested that MUC1 and MUC16 are not significantly altered in endometrial epithelium compared with healthy controls and that their expression remains stable throughout the menstrual cycle [16]. Another study reported even a slight but significant decrease in MUC1 in endometriotic lesions [19], while elevated MUC1 was described in lesions of ovarian endometriosis [20]. Particularly, MUC1, normally expressed by epithelial cells of the endometrium, was identified to be a marker of endometrial glands outside the uterus and discussed to have immunogenic properties which unfold during disease progression [20]. Accordingly, our findings in ectopic lesions indicate that mucin dysregulation is a hallmark of endometriotic tissue rather than of eutopic endometrium, pointing to a disease-specific alteration in the ectopic microenvironment.

Gene expression data from the EndometDB confirmed a complex regulation pattern for mucins, with *MUC1* transcript levels significantly increased in lesions relative to control peritoneum, yet still lower than in healthy endometrium. The latter could be explained by the fact that the relative amount of ectopic endometrial epithelial cells, infiltrating the respective tissue (peritoneum, ovaries, etc.) and normally expressing MUC1, are comparatively lower in the lesions than in endometrium. Interestingly, protein expression of *MUC6* and *MUC16* was elevated in lesion tissue, although transcript levels were not significantly different, suggesting regulation at post-transcriptional or post-translational levels, which has already been widely discussed for mucins in various tissues [21,22]. In addition to post-transcriptional regulation, several characteristics of mucin biology and the inflammatory microenvironment of endometriosis lesions may contribute to this discrepancy between mRNA and protein levels [22]. Mucins are heavily *O*-glycosylated proteins, and this extensive glycosylation confers high structural stability and resistance to proteolytic degradation, allowing mucin proteins to accumulate at the tissue level independently of transcriptional upregulation [10]. Such protein–transcript discordance has been well described for mucins in chronic inflammatory conditions, where altered glycosylation and reduced turnover result in prolonged mucin persistence at epithelial surfaces [14,15]. Moreover, the inflammatory milieu of endometriosis lesions, characterized by elevated cytokines and dysregulated protease activity, may further affect mucin trafficking, intracellular retention, and degradation without directly altering gene expression. This is particularly relevant for MUC16, which undergoes proteolytic cleavage near the cell surface, releasing its extracellular domain (measured clinically as CA 125) while retaining a stable membrane-associated fragment. Altered shedding or cleavage dynamics in endometriosis could therefore lead to increased tissue-associated MUC16 protein despite unchanged transcript levels and unaffected circulating CA 125 concentrations. These processes occur within the broader context of endometriosis pathogenesis, which is considered a multifactorial disease characterized by chronic inflammation, immune dysregulation, aberrant epithelial behavior, and estrogen-dependent signaling rather than retrograde menstruation alone [23,24,25,26]. Together, these mechanisms suggest that enhanced protein stability, altered inflammatory turnover, and modified shedding rates likely contribute to the elevated MUC6 and MUC16 protein abundance observed in endometriotic lesions [27,28]. Taken together, these mechanisms suggest that the elevated MUC6 and MUC16 protein levels observed in endometriotic lesions likely reflect a combination of enhanced protein stability, altered inflammatory turnover, and modified shedding dynamics rather than direct transcriptional upregulation alone.

Immunohistochemical analysis provided spatial context to these findings, revealing strong mucin immunoreactivity in epithelial cells of endometrial glands across multiple lesion subtypes. MUC1 was consistently elevated in pEM, OEM, and DIE, while MUC5AC expression was specifically enriched in peritoneal lesions. MUC2 was more heterogeneous and particularly variable in ovarian lesions. Spearman correlation analysis, as shown in Supplementary Table S1, revealed no significant association between menstrual cycle phase and MUC2 expression (r = 0.356, *p* = 0.161), nor between hormone therapy and MUC2 expression (r = 0.230, *p* = 0.330), indicating that MUC2 levels are not significantly influenced by these factors. These observations are consistent with previous reports associating MUC2 polymorphisms with endometriosis susceptibility and infertility [8]. The pronounced increase in alcian blue-positive staining further indicated a general enrichment of acidic glycoproteins, suggesting that endometriotic epithelia may undergo a mucinous transformation that alters the biochemical composition of the extracellular matrix.

Despite the elevated expression of mucins in tissue biopsies, classical mucin-derived soluble tumor markers were not discriminatory in serum or peritoneal fluid. Neither CA 125 (soluble form of MUC16) nor CA 15-3 (soluble form of MUC1) showed significant differences between patients and controls, in line with prior studies demonstrating limited diagnostic accuracy of CA 125 for endometriosis [29]. It has to be noted that the control cohort consisted of patients with other benign gynecological diseases, such as non-endometriosis-related ovarian cysts, uterine fibroids, hydrosalpinx, pelvic pain, or infertility, which could already lead to a comparable higher baseline of both markers. Moreover, in particular for patients with OEM the sample size was small (N = 6), making it difficult to completely rule out CA 125 as a marker for OEM. Although several publications have reported elevated CA 125 levels in the serum and peritoneal fluid of patients with EM, these studies often included relatively small patient cohorts, and numerous other investigations have highlighted substantial variability and methodological limitations in these findings [30]. In clinical practice, CA 125 may indeed be elevated, particularly in cases of endometrioma, and such elevations often occur in patients with EM. However, CA 125 is not a validated biomarker for the disease: normal levels cannot exclude EM, and elevated levels are not specific enough to confirm it. The marker is well known to increase in a range of benign gynecological conditions, including other ovarian cysts, as well as in malignant contexts such as ovarian cancer, further limiting its diagnostic utility. While cut-off values for serum CA 125 have been proposed as potential diagnostic tools [31,32], our data confirm that such markers lack sufficient sensitivity and specificity, particularly in heterogeneous patient populations. Thus, mucin dysregulation appears to be restricted to the local lesion environment rather than being systemically reflected in circulating markers.

Lectin blotting further revealed alterations in glycosylation status during endometriosis, with increased core-1 *O*-glycans (Galβ1-3GalNAc, T-antigen) and slight alteration of α(1-2)-linked fucose in lesion tissue. The lack of a statistically significant difference could be due to two factors: firstly, the sample size is small, which is a limitation of this study. Secondly, the tissue biopsies analyzed contained also cells other than ectopic endometrial epithelial cells, possibly masking the glycosylation changes in the epithelial cell layer of the lesion. Nevertheless, both glycans are integral to epithelial glycocalyx composition and are implicated in cell–cell and cell–matrix interactions as well as implantation processes [18,33]. In addition, core 1 *O*-glycans have been found to serve as a targeting signal for MUC1 to accumulate at the apical membrane of epithelial cells [34]. Thus, the enrichment of the T-antigen in endometriotic lesions may reflect an adaptation that enhances adhesive and invasive properties of ectopic endometrial cells, which are rich in MUC1. However, it is known that altered glycosylation of MUC1, particularly T-, Tn-antigen and their sialylated forms, are increased on tumor cells and represents an antigenic motif of the transformed cells for immune cells [35], which could alter the recognition of ectopic endometrial cells during immunosurveillance. Previous reports of altered glycosylation patterns in endometrium and serum immunoglobulin G (IgG) of women with advanced disease [36,37,38] support this interpretation. Interestingly, α-linked GalNAc, which was significantly reduced in EM tissue sections with different disease stages in a study also using DBA lectin for detection [36], showed no consistent differences and wide inter-individual variation in EM lesions. Likewise, global sialic acid content was not markedly altered in lesions in the present study whereas contrary observations were reported in previous studies. Accordingly, using the same lectin, SNA in an enzyme linked immunosorbent assay (ELISA) and lectin blot approach, α-2,6 sialylation in both peritoneal fluid and primary endometrial and endometriotic cell lines together with the mRNA expression of the sialylating enzyme *ST6GALNAC1* was significantly reduced [39]. Also, measuring sialylation of IgGs with an SNA- and MAA- based ELISA, Sołkiewicz et al. reported significantly lower α-2,3- and α-2,6-linked sialic acid levels in serum of EM patients [37]. It could be possible that this reduction in sialic acid cannot be detected on the tissue level and would only be measurable in biofluids (serum, peritoneal fluid) or on the cellular level. Yet, also regarding the sialic level of cells contradictory results can be found in the literature, as an increase in α-2,6 sialylation, also detected with SNA and determined with lectin blots and flow cytometry, was observed in human endometrial cell lines [40]. This highlights the heterogeneity of glycosylation changes in endometriosis. Overall, an altered glycan pattern of mucins could also result in changes in the endometrial microbiome, as the mucin *O*-glycan composition is crucial for the interaction of microbiota and the host and determines which bacteria can accumulate [41]. Consequently, this dense coat of sugars shapes the microbiome thereby impacting the immune defense of the respective organ [42]. It was already observed that the endometrial microbiome in endometriosis patients was altered [43,44], thus further studies should decipher these alterations in connection with changes in the sugar composition.

Importantly, correlation analyses did not identify associations between mucin expression or glycosylation changes and clinical parameters such as age, BMI, rASRM stage, reproductive history, or pain symptoms. These findings underscore that, although mucins and their glycosylation patterns are clearly altered in endometriosis lesions, their expression does not straightforwardly correlate with disease burden. This may further reflect the multifactorial nature of endometriosis, where molecular alterations at the tissue level do not directly predict clinical manifestation. Nevertheless, our findings emphasize that mucins and their glycan modifications are consistently dysregulated within lesions, making them promising candidates for future diagnostic or therapeutic exploration at the tissue level.

Taken together, the results of our study establish mucins as key players in the pathophysiology of endometriosis. While their value as circulating biomarkers remains limited, their lesion-specific upregulation and altered glycosylation suggest functional contributions to adhesion, immune evasion, and persistence of ectopic endometrium. Future work should investigate whether targeting mucin biosynthesis or glycosylation pathways could provide novel strategies for therapy. In addition, more refined approaches integrating mucin expression with multi-omics and clinical phenotyping may uncover patient subgroups for whom mucin signatures hold predictive or prognostic value.

## 4. Materials and Methods

### 4.1. Antibodies and Lectins Used

The primary antibodies used in this study included mouse anti-MUC1/EMA (clone E29, M0613, Agilent Technologies, Santa Clara, CA, USA), rabbit anti-MUC2 (ab134119, Abcam), mouse anti-MUC6 (sc-33668, Santa Cruz Biotechnology, Dallas, TX, USA), mouse anti-MUC16/CA 125 (clone 5E1, MABC1608, Sigma-Aldrich, St. Louis, MO, USA), and mouse anti-MUC5AC (clone 45M1, MA5-12178, Thermo Fisher Scientific, Waltham, MA, USA) and rabbit antiserum MAN-5ACI for the polypeptides of MUC5AC (generous gift from Prof. Dr. David Thornton) [45]. In addition, several lectins were employed to detect specific glycan structures, including TRITC-labelled Dolichos biflorus agglutinin (DBA, GlycoMatrix™, Dublin, OH, USA), biotinylated Maackia amurensis lectin II (MAL II), biotinylated Sambucus nigra lectin (SNA), FITC-labelled wheat germ agglutinin (WGA, Sigma Aldrich), biotinylated peanut agglutinin (PNA), FITC-labelled Aleuria aurantia lectin (AAL), and FITC-labelled Ulex europaeus agglutinin I (UEA I), all obtained from Vector Laboratories, Inc. (Newark, NJ, USA). All antibodies and lectins were used following the manufacturers’ instructions, with concentrations and incubation times optimized for the specific experimental protocols.

### 4.2. Samples

This study consisted of three separate analyses, each involving different groups of women with and without endometriosis. For the immunohistochemistry staining, tissue samples were collected during laparoscopic surgery at Charité–Universitätsmedizin Berlin. Patients were selected based on clinical evaluations, intraoperative findings, and subsequent histopathological results. Control samples were obtained from women without endometriosis who underwent laparoscopy for benign gynecological conditions, such as non-endometriosis-related ovarian cysts, uterine fibroids, hydrosalpinx, pelvic pain, or infertility. A total of 36 samples were analyzed from patients with endometriosis, which included 10 cases of pEM, 10 cases of DIE, 11 cases of OEM and 5 eutopic endometria. Additionally, location-matched control samples were collected, totaling 21 controls (5 peritoneum, 3 bladders, 1 appendix, and 1 rectum, 5 ovaries, and 6 eutopic endometria).

The gene expression analysis used publicly available data comprising 115 patients with endometriosis and 53 control participants. Further details regarding sample types, methodologies, and clinical metadata are described in the relevant analysis sections.

For the analysis of CA 125 and CA 15-3 in serum and peritoneal fluid, samples were collected from women with and without endometriosis. Serum samples were obtained from patients with endometriosis before anesthesia induction on the day of laparoscopic surgery, as well as from outpatients diagnosed with endometriosis at Charité–Universitätsmedizin Berlin (N = 22). Control serum samples (N = 10) were sourced from the institutional blood donation bank or collected from healthy volunteers recruited via announcements on the Charité intranet and through personal networks. Peritoneal fluid samples were collected intraoperatively during laparoscopy from patients with endometriosis (N = 44) and from women without endometriosis (N = 18) who were undergoing surgery for benign gynecological conditions like ovarian cysts, uterine fibroids, hydrosalpinx, pelvic pain, or infertility. They were aspirated from the pouch of Douglas immediately after trocar insertion to minimize contamination with blood. All samples were immediately processed, aliquoted, and stored at −80 °C until analysis.

The characteristics of the patients involved in all the analyses are listed in Supplementary Table S3.

### 4.3. Immunochemical Mucin Analysis

All surgically excised lesions (from endometriosis patients) and healthy peritoneum, endometrium, bladder, appendix, rectum, and ovary (control samples) were immediately fixed in buffered formalin 4% for 12–24 h and thereafter embedded in paraffin. Sections of two µm-thickness were immunohistochemically stained with antibodies against highly *O*-glycosylated mucins: MUC1, MUC2, MUC5AC, MUC6, and MUC16.

Following dehydration in xylene and graded alcohols, antigen retrieval was conducted using citrate buffer at pH 6.0 in a steamer for 20 min. Subsequently, slides were permeabilized with 1% Triton-X-100 in TBS for 20 min, followed by a 1 h incubation with protein block. Primary antibody incubation occurred overnight, followed by incubations with biotinylated secondary antibody for 1 h, peroxidase-labelled streptavidin for 45 min, and diaminobenzidine for 7 min. Finally, counterstaining with hematoxylin for 10 s was performed.

Endometrium (MUC1, MUC16), colon (MUC2), and stomach sections (MUC5AC, MUC6) were used as positive control tissue. Negative control sections were prepared by excluding the specific primary antibody during processing. Staining was detected using KEYENCE BZ-X80 (Keyence GERMANY GmbH, Neu-Isenburg, Germany). Photomicrographs were taken at different magnifications (40×, 100×, 200×) and were further processed using FIJI ((Fiji Is Just) ImageJ, version 2.16.0/1.54p, NIH, Bethesda, MD, USA) [46].

The Immunoreactive Score (IRS) was employed to evaluate mucin staining according to a standardized scoring system. The IRS is calculated by multiplying the staining intensity, scored as 0 (no reaction), 1 (weak reaction), 2 (moderate reaction), or 3 (strong reaction), by the proportion of positively stained cells, which is scored as 0 (no staining), 1 (<10% of cells stained), 2 (10–50% stained), 3 (51–80% stained), or 4 (>80% stained). Based on the resulting IRS, staining was classified as negative (0–2), weakly positive (3–4), moderately positive (6–8), or strongly positive (9–12) [47]. The assessment was independently performed by two experienced pathologists, who evaluated and categorized both the intensity and proportion of mucin-positive cells to ensure accuracy and reproducibility.

### 4.4. Histochemical Glycoprotein Analysis

Histochemical detection of acidic glycoproteins was performed using alcian blue staining. Tissue sections were first deparaffinized and rehydrated through a graded ethanol series. alcian blue solution (pH 2.5) was applied to the sections to selectively stain acidic mucopolysaccharides and glycoproteins. After incubation for 5 min at room temperature, sections were rinsed thoroughly with distilled water. Nuclear counterstaining was carried out using Nuclear Red to visualize cell nuclei, providing morphological context to the glycoprotein distribution. Following staining, sections were dehydrated, cleared, and mounted with a permanent mounting medium. Stained slides were examined under a light microscope, and images were captured with the KEYENCE BZ-X810 (Keyence GERMANY GmbH, Neu-Isenburg, Germany) for qualitative and quantitative analysis.

### 4.5. Mucin and Glycan Analysis by Agarose Gel Western Blotting

Mucin and glycan profiling was performed using agarose gel Western blotting combined with lectin detection to characterize specific glycan epitopes. Proteins separated by agarose gel electrophoresis were transferred onto membranes and subsequently either probed with antibodies for human mucins MUC1, MUC2, MUC5AC, MUC6, MUC16 or with lectins recognizing distinct glycan structures. α(2-3)-linked N-acetylneuraminic acid (Neu5Ac) residues were detected using MAL II, while α(2-6)-linked Neu5Ac residues were identified with SNA. β(1-4)-linked N-acetyl-D-glucosamine (GlcNAc) was detected using WGA, and galactose-β(1-3)-N-acetyl-D-galactosamine (Gal-β(1-3)-GalNAc) was recognized by PNA. Fucosylated glycans were detected using AAL, which binds fucose residues either α(1-6)-linked to N-acetyl-D-glucosamine or α(1-3)-linked to N-acetyllactosamine, whereas UEA I specifically detected α(1-3)-linked fucose. Lectin binding was visualized using the ChemiDoc-Imager (BioRad, Hercules, CA, USA) either via their fluorescence properties or using anti-biotin horseradish peroxidase (HRP) (#7075S, Cell Signaling Technology, Danvers, MA, USA) in combination with ECL kit from BioRad (#1705060) as secondary detection systems, while the respective HRP-coupled secondary antibody followed by ECL was used for mucin analysis, enabling detailed analysis of mucin glycosylation patterns.

### 4.6. Tumor Biomarker Quantification

Tumor markers CA 125 and CA 15-3 (glycosylation-dependent fragments of the mucins MUC16 and MUC1, respectively) were quantified in biological samples obtained from patients with and without endometriosis. CA 125 levels were measured in both serum and clear peritoneal fluid, whereas CA 15-3 was measured only in serum. All measurements were carried out at Labor Berlin using an electrochemiluminescence immunoassay (ECLIA) on the Roche Diagnostics platform (Indianapolis, IN, USA).

### 4.7. Gene Expression in Endometriosis Lesions, Peritoneum, and Endometrium

Gene expression of mucins in the tissue of EM patients and control groups was assessed/analyzed using data from the EndometDB Turku Endometriosis Database (https://endometdb.utu.fi/ (accessed on 3 July 2024)). This web-based data set contains gene expression data of over 24,000 genes from 115 EM patients and 53 controls [48]. Hormonal treatments taken within 3 months before surgery were documented during clinical examination and used to assign samples treated or untreated.

### 4.8. Statistical Analysis

All statistical analyses were performed using GraphPad Prism software, version 8.0.2 (GraphPad Inc., San Diego, CA, USA). Data were first assessed for normality using the Shapiro–Wilk test. For comparisons between two groups, an unpaired two-tailed t-test was applied for normally distributed data, while the Mann–Whitney U test was used for non-parametric data. For multiple group comparisons, the Kruskal–Wallis test followed by Dunn’s multiple comparison test was employed. Correlations between variables were assessed using the Spearman rank correlation test. Statistical significance was defined as *p* < 0.05.

## 5. Conclusions

This study demonstrates that mucin expression and glycosylation are consistently dysregulated in endometriotic lesions, with marked upregulation of MUC1 and MUC5AC in the endometrial gland region, in addition to overexpression of MUC2, MUC6, and MUC16 in EM biopsies and distinct alterations in glycan composition. While these changes were not reflected in circulating tumor markers such as CA 125 or CA 15-3, they highlight mucins as key contributors to the local pathophysiology of endometriosis. Our findings underscore the potential of mucin biology as a target for future diagnostic and therapeutic strategies, and they provide a foundation for integrating mucin profiles into patient-specific models of disease.

## Data Availability

The original contributions presented in this study are included in the article/Supplementary Materials. Further inquiries can be directed to the corresponding authors.

## Supplementary Materials

The following supporting information can be downloaded at https://www.mdpi.com/article/10.3390/ijms27021010/s1.

## Abbreviations

The following abbreviations are used in this manuscript:

DIE	Deep infiltrating endometriosis
OEM	Ovarian endometriosis
pEM	Peritoneal endometriosis
