# Supplementary material for: Mucin Biology as a Local Diagnostic and Promising Therapeutic Target in Endometriosis: Expression and Glycosylation Profiling
Source: Int J Mol Sci. 2026 Jan 20;27(2):1010. doi: 10.3390/ijms27021010 (PMC12842299; doi:10.3390/ijms27021010)
Supplement: Supplementary file 1 [file ijms-27-01010-s001.zip › ijms-4061327-supplementary.pdf]

Supplementary Material

Mucin Biology as a Local Diagnostic and Promising Therapeutic Target in Endometriosis: Expression and Glycosylation Profiling

Renata V. Velho[1], Christoph Schüßler[2], Lisa Strey[1], Stefanie Weigel[2], Susanne Thomsen[3], Franziska Ebert[2], Jonathan Pohl[4], Sylvia Mechsner[1,]* and Maria Maares[2,]*

1 Department of Gynecology Charité with Centre of Oncological Surgery, Endometriosis Research Centre Charité, Campus Virchow-Klinikum, Augustenburger Platz 1, 13353 Berlin, Germany; renata.voltolini-velho@charite.de (R.V.V.); lisa.strey@charite.de (L.S.)

2 Department of Food Chemistry, Institute of Nutritional Science, University of Potsdam, Karl-Liebknecht-Str. 24-25, 14476 Potsdam, Germany; christoph.schuessler@uni-potsdam.de (C.S.); stefanie.weigel@uni-potsdam.de (S.W.); fraebert@uni-potsdam.de (F.E.)

3 Department of Food Chemistry and Toxicology, Technische Universität Berlin, Strasse des 17. Juni 135, 10623 Berlin, Germany; susanne.thomsen@tu-berlin.de

4 Institute of Pathology, Charité-Universitätsmedizin Berlin, Charitéplatz 1, 10117 Berlin, Germany; jonathan.pohl@charite.de

*Correspondence: sylvia.mechsner@charite.de (S.M.); maria.maares.1@uni-potsdam.de (M.M.)

*Corresponding authors:
Maria Maares
Department of Food Chemistry, Institute of Nutritional Science, University of Potsdam, Karl-Liebknecht-Str. 24-25, 14476 Potsdam, Germany
Email: maria.maares.1@uni-potsdam.de
Phone: +49(0)331 977295508

Sylvia Mechsner
Department of Gynecology Charité with Centre of Oncological Surgery, Endometriosis Research Centre Charité, Campus Virchow-Klinikum, Augustenburger Platz 1, 13353 Berlin, Germany
Email: Sylvia.mechsner@charite.de
Phone: +49 030 450664866

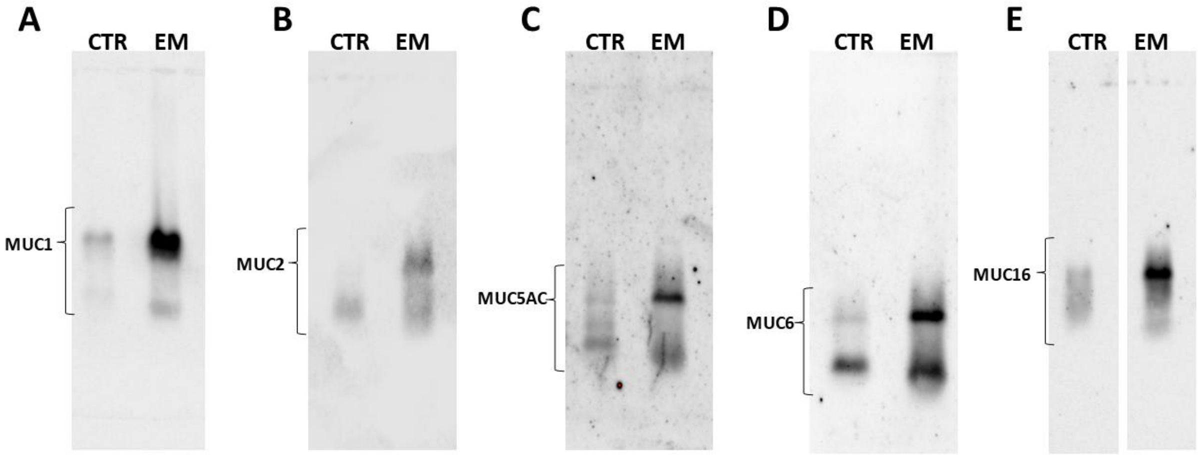

Supplementary Figure S1: Mucin agarose gel blots

Shown are representative blots of control (CTR) and endometriosis (EM) samples stained with antibodies against human MUC1 (A), MUC2 (B), MUC5AC (C), MUC6 (D), MUC16 (E).

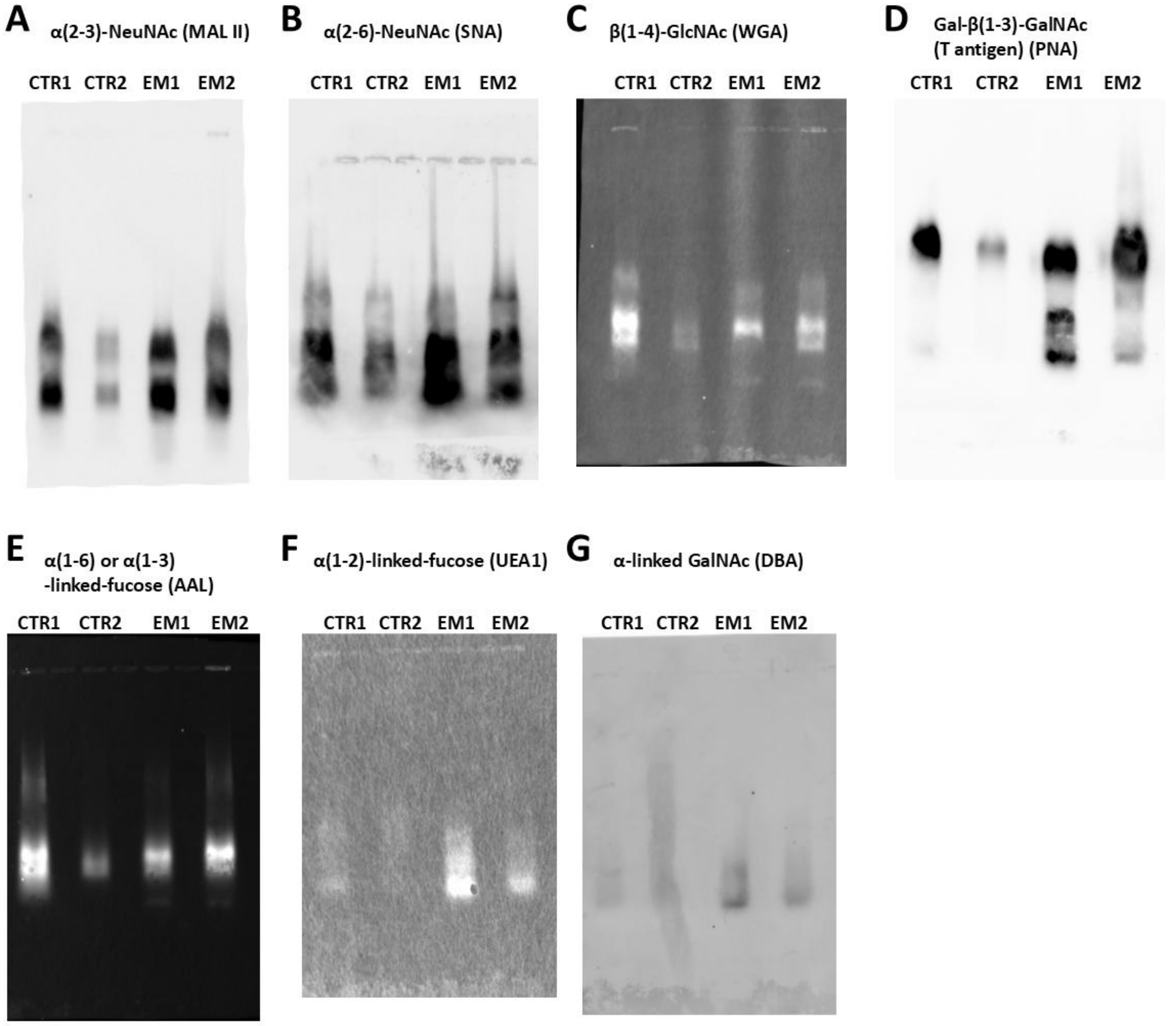

Supplementary Figure S2: Lectin agarose gel western blots

Shown are representative blots of control (CTR) and endometriosis (EM) samples stained with lectins against α(2-3)- N-acetylneuraminic acid (NeuNAc) (using Maackia Amurensis lectin (MAL) II) (A), NeuNAc in α(2-6)-linkage to terminal galactose (Sambucus Nigra lectin (SNA)) (B), β(1-4)-linked N-Acetal-D-glucosamin (GlcNAc) (wheat germ agglutinin (WGA)) (C), galactose in β(1-3)-linkage to N-Acetly-D-galactosamin (GalNAc) (peanut agglutinin (PNA)) (D), fucose in α(1-2) linkage (Ulex Europaeus agglutinin (UEA) I) (F), fucose either α(1-6) linked to N-Acetyl-D-glucosamine or α(1-3) linked to N-Acetyllactosamine (Aleuria Aurantia lectin (AAL)) (E), and α-linked GalNac (Dolichos Biflorus agglutinin (DBA)) (G).

Supplementary Table S1: Correlation analysis of immunochemical mucin staining and clinical data

		r- Value	*p*- Value
IRS MUC1	Age	r = 0.011	*p* = 0.964
	BMI	r = 0.013	*p* = 0.955
	EM type	r = 0.000	*p* = 1.000
	rASRM	r = - 0.104	*p* = 0.664
	Hormonal therapy	r = - 0.141	*p* = 0.553
	Menstrual cycle	r = - 0.154	*p* = 0.556
	Gravida	r = 0.000	*p* = 1.000
	Para	r = 0.000	*p* = 1.000
	KiWU	r = - 0.149	*p* = 0.530
IRS MUC2	Age	r = - 0.040	*p* = 0.866
	BMI	r = 0.086	*p* = 0.717
	EM type	r = 0.154	*p* = 0.518
	rASRM	r = 0.218	*p* = 0.356
	Hormonal therapy	r = 0.230	*p* = 0.330
	Menstrual cycle	r = 0.356	*p* = 0.161
	Gravida	r = 0.318	*p* = 0.172
	Para	r = 0.318	*p* = 0.172
	KiWU	r = - 0.134	*p* = 0.575
IRS MUC5AC	Age	r = - 0.105	*p* = 0.660
	BMI	r = - 0.365	*p* = 0.113
	EM type	r = - 0.304	*p* = 0.192
	rASRM	r = - 0.206	*p* = 0.383
	Hormonal therapy	r = 0.095	*p* = 0.690
	Menstrual cycle	r = 0.190	*p* = 0.465
	Gravida	r = - 0.149	*p* = 0.530
	Para	r = - 0.149	*p* = 0.530
	KiWU	r = - 0.074	*p* = 0.757
IRS MUC16	Age	r = 0.312	*p* = 0.180
	BMI	r = 0.161	*p* = 0.497
	EM type	r = 0.115	*p* = 0.628
	rASRM	r = 0.425	*p* = 0.062
	Hormonal therapy	r = 0.320	*p* = 0.170
	Menstrual cycle	r = 0.307	*p* = 0.230
	Gravida	r = 0.099	*p* = 0.678
	Para	r = 0.099	*p* = 0.678
	KiWU	r = - 0.138	*p* = 0.562

No correlation analysis could be obtained for MUC6, as this mucin was not expressed in the tissues and lesions tested. IRS: immunoreactive score; EM: endometriosis

Supplementary Table S2: Correlation analysis of tumor markers and clinical data

		***r*- Value**	***p*- Value**
MUC16/CA 125 levels in peritoneal fluid	EM type	r = -0.014	*p* = 0.910
	rASRM	r = 0.034	*p* = 0.791
	Hormonal therapy	r = 0.049	*p* = 0.699
	Menstrual cycle	r = 0.148	*p* = 0.240
	Dysmenorrhea	r = 0.021	*p* = 0.869
	Dyspareunia	r = -0.200	*p* = 0.111
	Dyschezia	r = -0.148	*p* = 0.239
	Dysuria	r = -0.044	*p* = 0.729
	Pelvic pain	r = -0.115	*p* = 0.363
	MUC16/CA 125 levels in serum	r = 0.183	*p = 0.393*
	MUC1/CA 15-3 levels in serum	r = -0.142	*p = 0.507*
MUC16/CA 125 levels in serum	EM type	r = 0.315	*p* = 0.133
	rASRM	r = 0.224	*p* = 0.293
	Hormonal therapy	**r = 0.427**	***p* = 0.038**
	Menstrual cycle	**r = 0.476**	***p* = 0.019**
	Dysmenorrhea	r = 0.008	*p* = 0.970
	Dyspareunia	r = -0.068	*p* = 0.111
	Dyschezia	r = -0.125	*p* = 0.560
	Dysuria	r = -0.047	*p* = 0.828
	Pelvic pain	r = -0.324	*p* = 0.122
	MUC1/CA 15-3 levels in serum	r = 0.118	*p = 0.582*
MUC1/CA 15-3 levels in serum	EM type	r = 0.059	*p* = 0.782
	rASRM	r = -0.035	*p* = 0.872
	Hormonal therapy	r = 0.129	*p* = 0.549
	Menstrual cycle	r = 0.090	*p* = 0.675
	Dysmenorrhea	r = -0.232	*p* = 0.275
	Dyspareunia	r = 0.137	*p* = 0.525
	Dyschezia	r = 0.094	*p* = 0.663
	Dysuria	r = -0.171	*p* = 0.424
	Pelvic pain	r = 0.164	*p* = 0.444

Supplementary Table S3: Characteristics of the patients involved in all the analyses

Immunohistochemistry staining			
		Patients	Controls
		n = 36	n = 21
**Age (years)**	Median	31	36
	Range	17 - 41	27 - 46
**EM Group**	pEM	10	/
	DIE	10	/
	OEM	11	/
	No grouping	5	/
**Stages (rASRM)**	I	7	/
	II	10	/
	III	10	/
	IV	8	/
	Missing data	1	/
**Menstrual cycle**	Menses	1	1
	Proliferative	12	3
	Secretory	11	6
	No cycle	3	1
	Missing data	9	10
**Hormons**	Yes	11	4
	Missing data	0	10
**BMI**	Underweight (<18.5)	3	0
	Normal (18.5 -24.9)	18	8
	Pre-Adipositas (25 – 29.9)	3	4
	Adipositas I (30 – 34.9)	1	3
	Adipositas II (35 – 39.9)	0	0
	Adipositas III (>40)	1	0
	Missing data	0	6
	Median	21.2	23.3
	Range	17.2 - 45.5	19.2 – 32.9
**Gene Expression Analysis**			
		Patients	Controls
		n = 330	n = 62
**Age (years)**	Median	32	38
	Range	20 - 48	27 - 48
**Tissue**	Endometrium	101	38
	Peritoneum	37	24
	pEM	76	/
	OEM	28	/
	DIE	88	/
**Stages (rASRM)**	I	39	/
	II	42	/
	III	76	/

	IV	166	/
	Missing data	7	/
**Menstrual cycle**	Menses	16	1
	Proliferative	50	10
	Secretory	75	19
	No cycle	150	22
	Missing data	39	10
**Hormons**	Yes	150	22
	No	170	40
**Tumor markers in serum**			
		Patients	Controls
		n = 22	n = 10
**Age (years)**	Median	31,5	41,5
	Range	23 - 47	24 - 48
**Stages (rASRM)**	I	6	/
	II	7	/
	III	4	/
	IV	3	/
	Missing data	2	/
**Menstrual cycle**	Menses	1	0
	Proliferative	10	1
	Secretory	10	0
	No cycle	0	0
	Missing data	1	9
**Hormons**	Yes	3	0
	Missing data	0	9
**Symptoms**	Dysmennorhea	20	/
	Dyschezia	13	/
	Dysuria	2	/
	Dyspareunia	18	/
	UBS	19	/
**Tumor markers in peritoneal fluid**			
		Patients	Controls
		n = 46	n = 18
**Age (years)**	Median	31	37
	Range	23 - 47	26 - 50
**EM Group**	OEM	8	/
	Other EM	38	/
**Stages (rASRM)**	I	10	/
	II	14	/
	III	9	/
	IV	11	/

	Missing data	2	/
**Menstrual cycle**	Menses	1	2
	Proliferative	21	8
	Secretory	22	6
	No cycle	0	0
	Missing data	2	2
**Hormons**	Yes	6	2
	Missing data	1	0
**Symptoms**	Dysmenorrhea	43	9
	Dyschezia	27	2
	Dysuria	8	2
	Dyspareunia	34	5
	UBS	39	13
